# The Effect of Screen-to-Screen Versus Face-to-Face Consultation on Doctor-Patient Communication: An Experimental Study with Simulated Patients

**DOI:** 10.2196/jmir.8033

**Published:** 2017-12-20

**Authors:** Kiek Tates, Marjolijn L Antheunis, Saskia Kanters, Theodoor E Nieboer, Maria BE Gerritse

**Affiliations:** ^1^ Department of Communication and Cognition Tilburg Center for Cognition and Communication Tilburg University Tilburg Netherlands; ^2^ Department of Obstetrics and Gynecology Radboud University Medical Center Nijmegen Netherlands; ^3^ Department of Obstetrics and Gynecology Gelderse Vallei Hospital Ede Netherlands

**Keywords:** teleconsultation, communication quality, patient satisfaction, provider satisfaction, information exchange, interpersonal relationship building, shared decision making

## Abstract

**Background:**

Despite the emergence of Web-based patient-provider contact, it is still unclear how the quality of Web-based doctor-patient interactions differs from face-to-face interactions.

**Objective:**

This study aimed to examine (1) the impact of a consultation medium on doctors’ and patients’ communicative behavior in terms of information exchange, interpersonal relationship building, and shared decision making and (2) the mediating role of doctors’ and patients’ communicative behavior on satisfaction with both types of consultation medium.

**Methods:**

Doctor-patient consultations on pelvic organ prolapse were simulated, both in a face-to-face and in a screen-to-screen (video) setting. Twelve medical interns and 6 simulated patients prepared 4 different written scenarios and were randomized to perform a total of 48 consultations. Effects of the consultations were measured by questionnaires that participants filled out directly after the consultation.

**Results:**

With respect to patient-related outcomes, satisfaction, perceived information exchange, interpersonal relationship building, and perceived shared decision making showed no significant differences between face-to-face and screen-to-screen consultations. Patients’ attitude toward Web-based communication (b=−.249, *P*=.02 and patients’ perceived time and attention (b=.271, *P*=.03) significantly predicted patients’ perceived interpersonal relationship building. Patients’ perceived shared decision making was positively related to their satisfaction with the consultation (b=.254, *P*=.005). Overall, patients experienced significantly greater shared decision making with a female doctor (mean 4.21, SD 0.49) than with a male doctor (mean 3.66 [SD 0.73]; b=.401, *P*=.009). Doctor-related outcomes showed no significant differences in satisfaction, perceived information exchange, interpersonal relationship building, and perceived shared decision making between the conditions. There was a positive relationship between perceived information exchange and doctors’ satisfaction with the consultation (b=.533, *P*<.001). Furthermore, doctors’ perceived interpersonal relationship building was positively related to doctors’ satisfaction with the consultation (b=.331, *P*=.003).

**Conclusions:**

In this study, the quality of doctor-patient communication, as indicated by information exchange, interpersonal relationship building, and shared decision making, did not differ significantly between Web-based and face-to-face consultations. Doctors and simulated patients were equally satisfied with both types of consultation medium, and no differences were found in the manner in which participants perceived communicative behavior during these consultations. The findings suggest that worries about a negative impact of Web-based video consultation on the quality of patient-provider consultations seem unwarranted as they offer the same interaction quality and satisfaction level as regular face-to-face consultations.

## Introduction

Nowadays, new information and communication technologies have an increasingly prominent role within medical practice [1], and the large majority of studies in this field reveal predominantly positive results of these new technologies, including improved quality and efficiency of health care, enhanced patient participation, access to a wider range of specialists, and time and cost savings [2-5]. Despite the apparent promise, large-scale implementation of Web-based consultations in health care has proven to be difficult, and several studies emphasize the continuing need for research on the impact of these developments on daily medical practice [5-8]. Furthermore, there is only a small amount of evidence indicating that Web-based patient-provider contact results in outcomes comparable or better than face-to-face care [9].

This contradicts the expectation that in the forthcoming years, Web-based consultation will increasingly replace the traditional face-to-face contact in patient-provider interactions [3,10,11]. Although Web-based screen-to-screen contact between patient and health provider (also referred to as video consultation, computer-mediated consultation, or teleconsultation) so far remains relatively uncommon, there is substantial evidence that patients want access to Web-based communication with health care providers adjacent to the regular face-to-face consultations [3,6,7]. Recent studies show that patients hold more positive attitudes concerning Web-based consultations as an acceptable medium for patient-provider communication than health professionals do [6,11-13]. In general, providers seem to be more hesitant, as they are concerned that Web-based consultations are lower in quality than offline consultations and might lead to a depersonalization of health care [3,6,14,15], although recent studies have found that physicians with previous electronic health (eHealth) experience show a more positive attitude to its implementation [7,8].

Further exploration of satisfaction with Web-based consultations is needed from the perspective of both patients and providers [16]. The quality and effectiveness of Web-based consultations may depend on patients’ and providers’ attitudes regarding Web-based communication. Therefore, this study seeks to investigate the impact of the medium of the consultation (screen-to-screen vs face-to-face provider-patient communication) on patients’ and providers’ communicative behavior and satisfaction with the consultation. Hence, our first 2 hypotheses read as follows:

H1a: Patients’ satisfaction with the consultation is higher in Web-based video consultations than in face-to-face consultations.

H1b: Providers’ satisfaction with the consultation is higher in face-to-face consultations than in Web-based video consultations.

Long-standing research of face-to-face consultations has shown that the 3 main pillars of medical communication (information exchange, interpersonal relationship building, and shared decision making) contribute to better patient-provider interactions and more satisfied patients and providers in offline consultations [17-19]. It is still unclear whether Web-based and face-to-face patient-physician interactions differ in quality in terms of information exchange, interpersonal relationship building, and shared decision making, and whether the mediating role of these communicative behaviors on patient and provider satisfaction differs.

Regarding information exchange, studies on computer-mediated communication may explain the impact of audiovisual Web-based consultation on the participants’ communicative behavior. Due to the reduction of visual and contextual cues in audiovisual Web-based communication, people tend to ask more questions and share more information in Web-based than in face-to-face settings [20,21]. In addition, we expect that the predictive value of communicative behavior on both patients’ and providers’ satisfaction with the consultation [18,19] will be generalizable to Web-based consultations. Therefore, our next hypotheses read as follows:

H2a: Web-based video consultations result in more information exchange than face-to-face consultations.

H2b: There will be a positive effect of information exchange on patients’ and providers’ satisfaction with the consultation.

As for interpersonal relationship building, there is no consensus yet whether Web-based patient-provider consultations are suitable for interpersonal relationship building. Although general studies on computer-mediated communication (CMC) have shown that audiovisual Web-based communication may be equally, or even more, suitable for affective interactions as face-to-face communication [21], empirical studies on patient-provider communication show mixed results [22]. However, in line with earlier research [21], we expect that providers will use more verbal statements of empathy in Web-based consultations to compensate for the lack of nonverbal empathy. Thus, patients’ self-disclosure will be likely to rise, leading to more or better interpersonal relationship building. Therefore, our next hypotheses state:

H3a: Web-based video consultations result in more interpersonal relationship building than face-to-face consultations.

H3b: There will be a positive effect of interpersonal relationship building on patients’ and providers’ satisfaction with the consultation.

**Figure 1:**
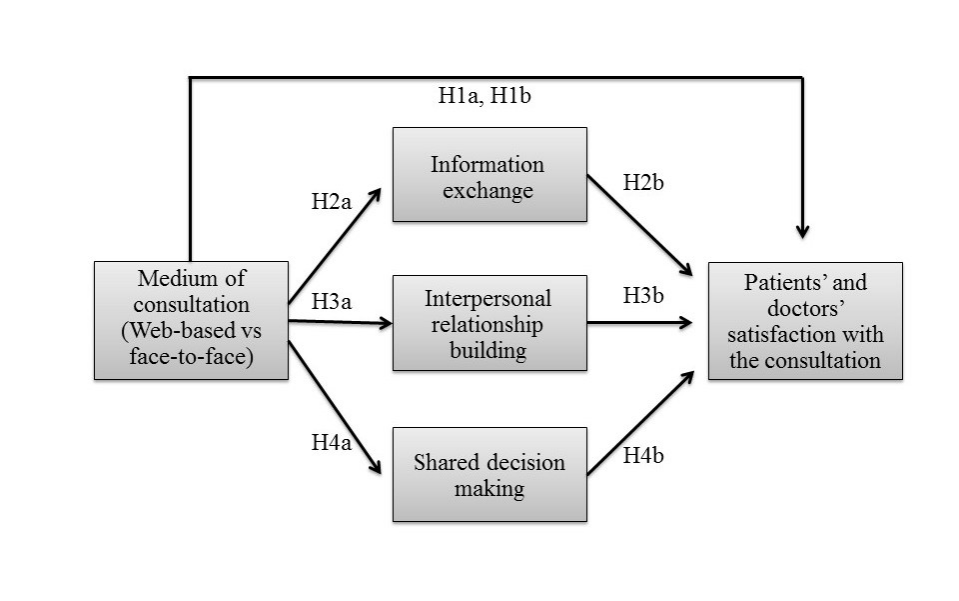
Overview of the hypotheses.

Over the last two decades, there has been a shift in support away from a paternalistic model of clinical decision making toward an approach wherein the patient takes a more active role, and decisions are reached in partnership between patient and provider [23]. Evidence has been found that the use of new communication and information technologies in health care fosters this paradigm shift toward increased patient participation and autonomy [11,24]. The positive effect of shared decision making on enhancing patients’ and providers’ satisfaction with the consultation has been well documented in face-to-face patient-provider communication [23,25]. Little evidence exists regarding the outcomes of shared decision making in Web-based consultations. It is expected that this predictive value also holds true for Web-based patient-provider communication, as stated in the following hypotheses:

H4a: Web-based video consultations result in more shared decision making than face-to-face consultations.

H4b: Both in Web-based and face-to-face consultations, there will be a positive effect of shared decision making on patients’ and providers’ satisfaction with the consultation.

Figure 1 presents an overview of the hypotheses formulated in this study.

## Methods

### Procedure

In our experiment, medical interns and simulated patients performed doctor-patient consultations on pelvic organ prolapse and urinary stress incontinence. These subjects were chosen because of the high prevalence in women: in Western countries, 20% of women would have undergone surgery for urinary incontinence or pelvic organ prolapse [26].

Twelve interns (fifth and sixth year medical students from the Radboud University Nijmegen in the Netherlands) participated in the experiment. Compared with medical doctors, interns have less experience with either face-to-face or Web-based consultations, which reduces the likelihood that there is an established preference for one or the other. Six certified simulated patients, who were trained to act as actual patients by simulating a set of symptoms [27], participated in the experiment. The use of simulated patients is an often used method, ensuring high experimental control over the conditions [28,29].

Four different scenarios (2 different scenarios with 2 conditions) on pelvic organ prolapse and urinary stress incontinence were written by 2 gynecologists from the Radboud University Medical Center (MG and TN). In the Netherlands, according to the ruling of the Dutch Healthcare Authority NZA [30], it is not allowed to have a first consultation on the Web, so the scenarios served as a second consultation. In the fictional first consultation, the patient had discussed her complaints with the doctor, a physical examination had taken place, and the patient had already been given her diagnosis. In the simulated consultation, the doctor explained the different treatment options, and a decision had to be made about the treatment plan.

Both scenarios for interns and simulated patients consisted of a summary of the first consultation, demographic details of the patient, and information about lifestyle, job, and children. Additional medical information about the diagnosis was added to the interns’ scenarios and a description of the concerns of the patient regarding her illness and the possible treatment, and preferences for certain treatment options were added to the scenarios of the simulated patients.

Each intern received all 4 scenarios. In addition, they received information about the treatment options. Treatment for pelvic organ prolapse can consist of lifestyle change, specialized physiotherapy, fitting of a vaginal pessary or surgery [31]. Two weeks after the interns received all information, a preparatory meeting with all interns was held with MG and SK, to answer any remaining questions. After the meeting, the interns received a questionnaire that they had to fill out directly after every consultation. Reading the questionnaire only after the first consultation could induce specific communicative behavior as described in the questions. With providing the questionnaire beforehand, the possible effect of priming was expected to be the same in all consultations.

Before the experiment, simulated patients received their scenarios and studied them. Later, a preparatory meeting with the simulated patients was held together with MG (gynecologist) and SK. In this meeting, all remaining questions regarding the scenarios and the experiment were answered, and each simulated patient was given the opportunity to practice one of the scenarios. In addition, they also received the questionnaire that they had to fill out directly after the actual experiment.

A total of 48 simulated doctor-patient consultations were held, of which 24 took place in the Web-based setting and the other 24 in the face-to-face setting. There were 6 experimental consultation sessions; 2 simulated patients and 2 interns participated in each session. Interns participated in 1 consultation session, whereas simulated patients participated in 3 consultation sessions. During each session, they each participated in 4 consultations, which were randomly divided over the 2 scenarios and 2 conditions in a manner that each intern and each simulated patient participated in 2 Web-based and 2 face-to-face consultations (see Multimedia Appendix 1). An intern and a simulated patient could not have a consultation together, right after they participated in a joined consultation. Finally, the study had a counterbalanced repeated measures design in which all scenarios were equally divided over the conditions and in which the order of the scenarios was randomized as well.

Participants in the audiovisual screen-to-screen-condition were led to separate rooms that were equipped with a laptop and a webcam with a built-in microphone, so they could both see and hear each other. The intern and patient interacted through Microsoft Skype. The intern would initiate a video call with the patient through Skype, after which the consultation began. There was no minimum length of the consultation; however, after 15 min, the participants were asked to finalize the consultation. After the consultation, both the intern and simulated patient answered the questionnaire about the consultation.

The face-to-face condition took place in a room that resembled a doctor’s office. The patient was led to the doctor’s office, after which the consultation began. After the consultation, the patient entered a separate room, so that both participants could fill out the questionnaire about the consultation separately. Care was taken, when switching between consultations, to avoid interns and simulated patients meeting each other right after a consultation. Multimedia Appendix 1 shows a flowchart of the study design per experimental consultation session.

### Sample

All interns were aged between 22 and 26 years (mean 23.8, SD 1.3). Both male (n=4) and female (n=8) interns participated in the experiment, as gender composition of a group may affect the interaction, such as the amount or type of self-disclosure. Only 1 intern had no prior experience with audiovisual Web-based communication, whereas the other interns reported to use applications for Web-based communication about once every 6 months (4), once every month (2), once every week (4), or more days per week (1), all for personal reasons. None of the interns had used audiovisual Web-based consultation to communicate with a patient.

The simulated patients, all female, were aged between 39 and 57 (mean 47.3, SD 6.3), to match the scenarios used in the experiment. All simulated patients were highly educated; they graduated from either higher vocational education or a university. Half of the simulated patients reported to use software for audiovisual Web-based communication, such as Skype or Facetime, about once every 6 months, whereas the other half of the patients had no prior experience with audiovisual Web-based communication. None of the participants had communicated with a doctor through Web-based consultation before.

### Measures

The operationalization of the mediating variables used in this study was based on the *Patient Participation Scale* (PPS) [32] and the *LEAPS Framework* (Listen, Educate, Assess, Partner and Support) [33] as the 3 main purposes of communication between doctors and patients during a consultation (ie, information exchange, interpersonal relationship building, and shared decision making) are best covered with these 2 scales. In Multimedia Appendix 2, the operationalization is described in more detail.

#### Perceived Information Exchange

Five items were used to measure patients’ perceived information exchange based on the PPS [32] and the LEAPS Framework [33]. Examples of the items can be found in the detailed version of the operationalization in Multimedia Appendix 2. The response categories for all items ranged from 1 (*completely disagree*) to 5 (*completely agree*). All items formed a one-dimensional scale (explained variance 50%, alpha=.73, mean 3.49 [SD 0.58]).

Doctors’ perceived information exchange was measured by 3 items from PPS as in the patients’ questionnaire [32], adapted to fit the doctors’ perspective. In addition, the following 3 items from the subparts information exchange and *identification of problems and concerns* of the LEAPS Framework [33] were added: “The patient had difficulty remembering instructions,” “The patient did not understand my explanations of the medical problem and treatment,” and “I could not understand all the patient wanted to tell me.” The 6 items (see Multimedia Appendix 2) formed a one-dimensional scale (explained variance 45%, alpha=.73, mean 4.13 [SD 0.41]).

#### Perceived Interpersonal Relationship Building

To measure patients’ perceived interpersonal relationship building, 5 items were used, which were derived from the subpart *patients’ evaluation of emotional support of the physician* from the Cologne Patient Questionnaire [34,35] and the subpart *interpersonal rapport* of the LEAPS Framework [33]. Examples can be found in the detailed version of the operationalization in Multimedia Appendix 2. Response categories ranged from 1 (*completely disagree*) to 5 (*completely agree*). The items formed a one-dimensional scale (explained variance 50%, alpha=.73, mean 3.80 [SD 0.49]).

To measure doctors’ perceived interpersonal relationship building, 5 items were used, which were derived from the Cologne Patient Questionnaire [34,35] and the subpart interpersonal rapport of the LEAPS Framework [33]. Response categories ranged from 1 (*completely disagree*) to 5 (*completely agree*). The items (see Multimedia Appendix 2) formed a one-dimensional scale (explained variance 47%, alpha=.67, mean 3.84 [SD 0.39]).

#### Perceived Shared Decision Making

Patients’ shared decision making was measured using 7 items: 3 items of PPS [32] and 4 items of the Cologne Patient Questionnaire [34,35]. Examples of the items can be found in Multimedia Appendix 2. Response categories ranged from 1 (*completely disagree*) to 5 (*completely agree*). The items formed a one-dimensional scale (explained variance 64%, alpha=.89, mean 4.02 [SD 0.63]).

The items measuring patients’ shared decision making were adapted to measure doctors’ shared decision making by turning the patient’s perspective to the doctor’s perspective. Example item is as follows: “I sufficiently involved the patient in decisions about the treatment.” The 7 items (see the examples in Multimedia Appendix 2) formed a one-dimensional scale (explained variance 54%, alpha=.83, mean 4.08 [SD 0.49]).

#### Satisfaction With the Consultation

Patients’ satisfaction with the consultation was measured using the *Patient Satisfaction Questionnaire* (PSQ) [36,37]. Items included were as follows: “How satisfied are you with the way the doctor addressed your needs?” All items were answered on a scale from 1 (*not at all satisfied*) to 5 (*extremely satisfied*). The 6 items formed a one-dimensional scale (explained variance 53%, alpha=.85, mean 4.33 [SD 0.42]).

To measure doctors’ satisfaction with the consultation, the items of *PSQ* and the item measuring satisfaction with the treatment decision were adapted to the doctors’ situation as suggested by Zandbelt and colleagues [38]. For example, the item “How well did the doctor address your needs?” was modified to “How well did you address the needs of this patient?” The 6 items formed a one-dimensional scale (explained variance 48%, alpha=.76, mean 4.29 [SD 0.39]).

#### Covariates

##### Attitude Toward Web-Based Communication

Doctors’ and patients’ attitude toward Web-based communication was measured with 6 items from Yen and Tu’s Revised *Computer-Mediated Communication Questionnaire* [39]. Items included the following: “CMC messages convey feeling and emotion,” “It is easy to express what I want to communicate through CMC,” and “My computer skills allow me to be comfortable while participating in CMC.” The response categories ranged from 1 (*completely disagree*) to 5 (*completely agree*). In both cases, the 6 items formed a one-dimensional scale. The doctors’ scale (explained variance 48%) had an alpha of .76 (mean 3.26, SD 0.52) and the patients’ scale (explained variance 44%) had an alpha of .92 (mean 3.17, SD 0.73).

##### Perceived Time and Attention

To measure patients’ perceived time and attention of the doctor, a total of 4 items were used from the LEAPS Framework [33] and Cologne Patient Questionnaire [34,35]. For example, “The doctor did not spend enough time with me” and “The doctor did not address all the problems I wanted to discuss.” The response categories ranged from 1 (*completely disagree*) to 5 (*completely agree*). The items formed a one-dimensional and reliable scale (explained variance 54%, alpha=.70, mean 3.81 [SD 0.58]).

### Statistical Analysis

To test the hypotheses, Preacher and Hayes’ procedure [40] to test indirect effects in multiple mediator models was used. Preacher and Hayes’ approach is similar to Baron and Kenny’s causal steps approach [41] in two respects. First, it also uses regression analyses to investigate how the independent variable (medium of consultation) influences the mediating variables (perceived information exchange, perceived interpersonal relationship building, and perceived shared decision making) and how the mediating variable influences the dependent variable (satisfaction with the consultation). Second, it also tests whether the influence of the independent variables on the dependent variable disappears when the mediating variable is included.

However, Preacher and Hayes’ approach extends Baron and Kenny’s causal steps approach, as with this procedure, multiple mediators can be tested simultaneously, which allows testing the effects of each single mediator while controlling for the effect of the other mediators, which in turn is particularly useful in this study. Furthermore, covariates can be considered. Finally, the approach of Preacher and Hayes [40] uses bootstrapping to test the significance of the mediating effects. This eliminates the need for multivariate normality, which is unlikely to be achieved in small samples. The analyses and bootstrap estimates that follow are based on 10,000 bootstrap samples. Hence, for each of the possible mediated effects, we tested first, based on the normal theory, whether the various paths that constitute our model were significant. Subsequently, we performed a formal test of the mediated effect based on the bootstrap method.

## Results

### Patient-Related Outcomes

**Figure 2:**
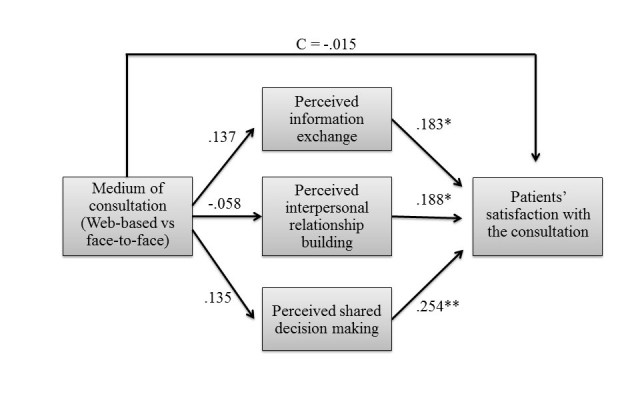
Summary of the patient-related results. N=46; R²=.69; *P<.05; **P<.01.

Two separate mediation analyses were conducted, one for the simulated patient-related outcomes and one for doctor-related outcomes. The first analysis was focused on the patients and compared patients’ satisfaction with the consultation between the Web-based video condition and the face-to-face condition (see Figure 2). The mediators used in the analysis were patients’ perceived information exchange, patients’ perceived interpersonal relationship building, and patients’ perceived shared decision making. The scenario used in the consultation, patients’ attitude toward Web-based communication, patients’ perceived time and attention, and doctors’ gender were entered as covariates.

Hypothesis 1a stated that satisfaction with the consultation would be higher in Web-based video consultations than in face-to-face consultations. The results showed no significant difference in patients’ satisfaction between the Web-based condition (mean 4.32, SD 0.41) and the face-to-face condition (mean 4.33 [SD 0.43], b=−0.015, Standard error [SE]=0.075, *P*=.85), which implies that the medium of consultation does not have an impact on patients’ satisfaction with the consultation. This rejects Hypothesis 1a.

Next, the 3 mediators were tested. Hypothesis 2a stated that *Web-based* video consultations result in more information exchange than face-to-face consultations. This hypothesis was rejected as patients’ perceived information exchange did not significantly differ between the Web-based condition (mean 3.54, SD 0.40) and the face-to-face condition (mean 3.43 [SD 0.71], b=.137, SE=0.162, *P*=.40). An analysis of the covariates showed that the scenario used in the consultation had an influence on patients’ perceived information exchange (b=.175, SE=0.074, *P*=.02), which indicates that some of the scenarios induce better or more information exchange than others.

Hypothesis 2b stated that information exchange would increase patients’ satisfaction with the consultation. This hypothesis was confirmed as the results showed a positive relationship between perceived information exchange and patients’ satisfaction (b=.183, SE=0.073, *P*=.02), which implies that more perceived information exchange leads to higher satisfaction with the consultation. Finally, the mediating effect of patients’ perceived information exchange was not significant (point estimate=.025, SE=0.034, 95% bias corrected and accelerated (Bca) CI −0.025 to 0.115).

Hypothesis 3a, which stated that Web-based video consultations would result in more interpersonal relationship building than face-to-face consultations, was not supported. Patients’ perceived interpersonal relationship building did not significantly differ between the Web-based condition (mean 3.75, SD 0.52) and the face-to-face condition (mean 3.84 [SD 0.48], b=−.058, SE=0.135, *P*=.67). An analysis of the covariates showed that patients’ attitude toward Web-based communication (b=−.249, SE=0.099, *P*=.02) and patients’ perceived time and attention (b=.271, SE=0.124, *P*=.03) significantly predicted patients’ perceived interpersonal relationship building. Hence, the more time or attention patients felt was spent on them, the better they rated the interpersonal relationship with their doctor. Patients’ attitude toward Web-based communication was negatively related to patients’ perceived interpersonal relationship building, which means that a lower attitude toward Web-based communication leads to more perceived interpersonal relationship building. When splitting the two conditions, the effect of patients’ attitude toward Web-based communication remained visible only in the screen-to-screen condition. Hypothesis 3b stated that interpersonal relationship building would increase patients’ satisfaction with the consultation. As expected, patients’ perceived interpersonal relationship building was positively related to patients’ satisfaction (b=.183, SE=0.088, *P*=.04), which confirms H3b. The mediating effect of patients’ perceived interpersonal relationship building was not significant (point estimate=−.011, SE=0.029, 95% Bca CI −0.084 to 0.034).

Hypothesis 4a, which stated that Web-based video consultations would result in more shared decision making than face-to-face consultations, was not supported. Patients’ perceived shared decision making did not significantly differ between the Web-based condition (mean 4.01, SD 0.57) and the face-to-face condition (mean 3.99 [SD 0.70]), b=.135, SE=0.133, *P*=.32). The analysis of the covariates showed that patients experienced significantly more shared decision making with a female doctor (mean 4.21, SD 0.49) than with a male doctor (mean 3.66 [SD 0.73], b=.401, SE=0.146, *P*=.009). In addition, patients’ perceived shared decision making was positively related to patients’ perceived time and attention (b=.604, SE=0.122, *P*<.001). Hypothesis 4b, which stated that shared decision making would increase patients’ satisfaction with the consultation, was confirmed. Patients’ perceived shared decision making was positively related to their satisfaction with the consultation (b=.254, SE=0.086, *P*=.005). There was no significant mediating effect of patients’ perceived shared decision making (point estimate=.034, SE=0.037, 95% Bca CI −0.024 to 0.121).

Finally, it was tested which covariates were significantly related to patients’ satisfaction with the consultations. The analysis showed that patients were significantly more satisfied with the consultation when they had communicated with a female doctor (mean 4.48, SD 0.31) than with a male doctor (mean 4.02 [SD 0.45], b=.259, SE=0.090, *P*=.007). For the covariates scenario of the consultation, patients’ attitude toward Web-based communication, and patients’ perceived time and attention, there was no direct effect on patients’ satisfaction with the consultation, with all *P* s>.15. Figure 2 provides a summary of the patient-related results. Path coefficients represent unstandardized regression weights, where C is the direct effect of the experimental condition on patients’ satisfaction with the consultation after inclusion of the mediators.

### Doctor-Related Outcomes

**Figure 3:**
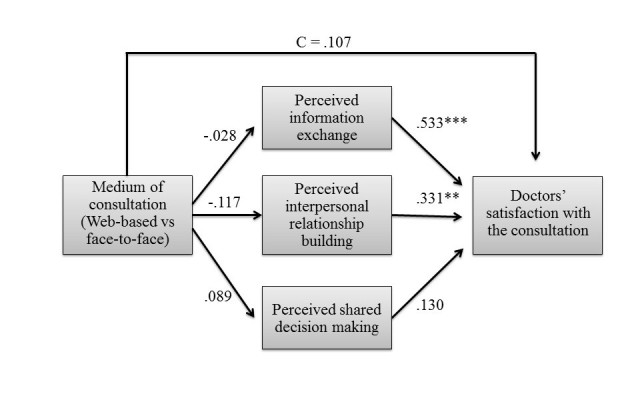
Summary of the doctor-related results. N=46; R²=.71; *P<.05; **P<.01, ***P<.001.

The second analysis compared doctors’ satisfaction with the consultation between the Web-based condition and the face-to-face condition (see Figure 3). The mediators used in the analysis were doctors’ perceived information exchange, doctors’ perceived interpersonal relationship building, and doctors’ perceived shared decision making. Doctors’ gender, the scenario used in the consultation, and doctors’ attitude toward Web-based communication were entered as covariates.

Hypothesis 1b stated that doctors’ satisfaction with the consultation would be higher in face-to-face consultations than in Web-based consultations. However, the results showed no significant difference in doctors’ satisfaction with the consultation between the Web-based condition (mean 4.31, SD 0.37) and the face-to-face condition (mean 4.27 [SD 0.41], b=.107, SE=0.066, *P*=.12), when examining the direct effect of the experimental condition on doctors’ satisfaction. This indicates that the medium of consultation did not have an impact on doctors’ satisfaction with the consultation. Therefore, Hypothesis 1b is rejected.

Next, the three mediators were tested. Hypothesis 2a, which stated that Web-based consultations result in more information exchange than face-to-face consultations, was rejected. Doctors perceived information exchange did not significantly differ between the screen-to-screen condition (mean 4.12, SD 0.42) and the face-to-face condition (mean 4.15 [SD 0.39], b=−.028, SE=0.121, *P*=.82). Hypothesis 2b stated that information exchange increases doctors’ satisfaction with the consultation. This hypothesis was confirmed as the results showed a positive relationship between perceived information exchange and doctors’ satisfaction with the consultation (b=.533, SE=0.093, *P*<.001), which implies that better perceived information exchange leads to higher satisfaction with the consultation. The mediating effect of doctors’ perceived information exchange was not significant (point estimate=−.015, SE=0.065, 95% Bca CI −0.143 to 0.113).

Hypothesis 3a, which stated that Web-based video consultations result in more interpersonal relationship building than face-to-face consultations, was not supported. Doctors’ perceived interpersonal relationship building did not significantly differ between the Web-based condition (mean 3.78, SD 0.36) and the face-to-face condition (mean 3.90 [SD 0.42], b=−.117, SE=0.107, *P*=.28). Hypothesis 3b, which stated that interpersonal relationship building would increase doctors’ satisfaction with the consultation, was confirmed. As expected, doctors’ perceived interpersonal relationship building was positively related to doctors’ satisfaction with the consultation (b=.331, SE=0.103, *P*=.003). The mediating effect of doctors’ perceived interpersonal relationship building was not significant (point estimate=−.039, SE=0.039, 95% Bca CI −0.079 to 0.023).

Hypothesis 4a, which stated that Web-based consultations would result in more shared decision making than face-to-face consultations, was not supported. Doctors’ perceived shared decision making did not significantly differ between the screen-to-screen condition (mean 4.04, SD 0.53) and the face-to-face condition (mean 4.13 [SD 0.46], b=−.089, SE=0.146, *P*=.54). Hypothesis 4b, which stated that shared decision making would increase doctors’ satisfaction, was rejected as well. Doctors’ perceived shared decision making was not significantly related to their satisfaction with the consultation (b=.130, SE=0.080, *P*=.11). Therefore, there was no significant mediating effect of doctors’ perceived shared decision making (point estimate=−.012, SE=0.024, 95% Bca CI −.077 to .024). Finally, the covariates were not related to the mediating variables or doctors’ satisfaction with the consultation, with all *P* s>.137. Figure 3 provides a summary of the doctor-related results. Path coefficients represent unstandardized regression weights, where C is the direct effect of the experimental condition on doctors’ satisfaction with the consultation after inclusion of the mediators.

### Post Hoc Analyses

The results of the mediation analyses showed no significant mediation with the medium of consultation (face-to-face vs Web-based). Therefore, post hoc analyses were performed to test whether satisfaction is indeed build on the same three communication pillars in Web-based consultations as in face-to-face consultations or that, maybe, a certain pillar is more important in one of the modes of communication. We examined whether the medium of consultation might perform as a moderator in the effect of the mediating variables on the dependent variable. First, the medium of consultation was examined as a moderator of the relation between patients’ perceived information exchange and patients’ satisfaction with the consultation. The medium of consultation explained a significant increase in variance in patients’ satisfaction with the consultation (Δ *R*^2^=.08, *F*_1,44_=4.06, *P*=.05), which implies that the effect of perceived information exchange on satisfaction depends on the medium of consultation. The effect of information exchange on satisfaction was only significant in the Web-based condition (b=.533, SE=0.207, *P*=.01), but not in the face-to-face condition (b=.054, SE=0.116, *P*=.65), suggesting that information exchange only predicts satisfaction when patients and doctors communicate online.

Second, it was examined whether the medium of consultation served as a moderator of the relation between patients’ perceived interpersonal relationship building and patients’ satisfaction with the consultation. The medium of consultation explained a significant increase in variance in patients’ satisfaction with the consultation (ΔR^2^=.10, F_1,44_=5.65, *P*=.02). Thus, the medium of consultation was a significant moderator of the relationship between patients’ perceived interpersonal relationship building and their satisfaction with the consultation. The effect of interpersonal relationship on satisfaction was only significant in the face-to-face condition (b=.545, SE=0.169, *P*=.002) but not in the Web-based condition (b=−.003, SE=0.156, *P*=.99), suggesting that interpersonal relationship building only predicts satisfaction when patients communicate face-to-face.

Third, it was examined whether the medium of consultation served as a moderator for the relation between patients’ perceived shared decision making and patients’ satisfaction with the consultation. The medium of consultation was not a significant moderator between shared decision making and satisfaction (ΔR^2^=.00, F_1,44_=.28, *P*=.60). The effect of shared decision making on satisfaction was significant in both the face-to-face condition (b=.482, SE=0.093, *P*<.001) and the Web-based condition (b=.404, SE=0.115, *P*=.001), which indicates that shared decision making predicts patients’ satisfaction with the consultation in both face-to-face and Web-based consultations.

Finally, the same analyses were performed to examine whether the medium of consultation served as a moderator between doctors’ perceived communicative behavior and doctors’ satisfaction with the consultations. None of the moderation models were significant, with all ΔR^2^<.02, all F<1.44, and all *P*>.235. This implies that the effect of doctors’ perceived information, doctors’ perceived interpersonal relationship building, and doctors’ perceived shared decision making on doctors’ satisfaction was not dependent on the medium of consultation, and was thus the same for face-to-face consultations and Web-based consultations.

## Discussion

### Effect of the Medium of Consultation on Communicative Behavior

In this study, we examined the difference in doctors’ and patients’ information exchange, interpersonal relationship building, and shared decision making between Web-based and face-to-face consultations. We found that there were no significant differences between Web-based and face-to-face consultations in any of these three communicative behaviors, which implies that doctors and simulated patients do not perceive communication during a consultation differently when communicating on the Web via video or face-to-face.

The finding that screen-to-screen consultations did not seem to differ from face-to-face consultations regarding the abovementioned outcomes is in line with the *Social Information Processing (SIP) theory* [42]. The *SIP theory* explains that to compensate for the reduction in nonverbal cues in Web-based consultations, doctors and patients may use more verbal cues to create or enhance interpersonal relationships and to exchange the same amount of information as they would face-to-face [42]. Doctors may thus be more likely to show empathy verbally in Web-based video interactions than in face-to-face interactions to compensate for the lack of nonverbal ways of doing so [21,43]. Their findings suggest that nonverbal cues, which are often seen as extremely important in face-to-face interactions [44], may be less important in Web-based interactions, because doctors and patients seem to find other ways to contextualize their messages and to gather and communicate essential information.

Furthermore, it is possible that doctors and simulated patients only *perceive* their communicative behavior the same way in Web-based and face-to-face consultations, whereas their actual communicative behavior differs. Previous research showed that doctors and patients have lower expectations from Web-based consultations than from face-to-face consultations [45]. Therefore, they may be less critical of shortcomings in communicative behavior during screen-to-screen interactions as compared with face-to-face consultations. The reduction of nonverbal cues and the lower level of social presence in Web-based communication may thus change doctors’ and patients’ expectations of the interaction and in turn their perception. This would imply that in Web-based consultations less cues are needed than in face-to-face consultations to achieve the same effect of perceived information exchange, perceived interpersonal relationship building, and perceived shared decision making.

### Satisfaction With Screen-to-Screen and Face-to-Face Consultations

A further aim of this study was to examine the mediating role of doctors’ and patients’ information exchange, interpersonal relationship building, and shared decision making on doctors’ and patients’ satisfaction with face-to-face and Web-based consultations. First, the results showed no difference between Web-based (screen-to-screen) and face-to-face consultations in doctors’ and simulated patients’ satisfaction with the consultation. Studies on regular (face-to-face) consultations have shown that communicative behavior is the most important predictor of doctors’ and patients’ satisfaction with the consultation [44,46]. Therefore, the fact that there was no difference in doctors’ and simulated patients’ perceived communicative behavior is most likely the explanation for the fact that doctors and simulated patients were as satisfied with the consultation when they communicated face-to-face online.

Second, it was examined whether the three main communicative behaviors of medical interactions had an impact on doctors’ and patients’ satisfaction. In line with earlier research on offline consultations [44,47,48], this study showed that both doctors’ and simulated patients’ perceived information exchange and perceived interpersonal relationship building are positively related to their satisfaction with face-to-face and Web-based consultations. This underlines the dual communicative needs of patients [49] as the findings indicate that the fulfillments of patients’ instrumental and emotional needs are both predictors of patients’ satisfaction with the consultation. In addition, perceived shared decision making was positively related to simulated patients’ satisfaction, which underlines earlier studies stating that patients want to play an active role in the decision-making process [50,51]. This in turn enhances their satisfaction with the decision about the treatment [52,53], which predicts general satisfaction with the consultation [54]. However, perceived shared decision making did not predict doctors’ satisfaction with the consultation. A possible reason for this could be that simulated patients experience shared decision making differently than doctors. Doctors may perceive shared decision making more as a form of information exchange and they may feel like they still have the final word about the treatment decision [55]. This was also observed in the study by Hamann et al [56], where it was found that if patients insist on their preferences and doubt their doctors’ recommendations, physicians consider it as less helpful and even become more annoyed.

The doctors’ gender was a significant predictor of simulated patients’ satisfaction with the consultation. Simulated patients were more satisfied after having a consultation with a female doctor than with a male doctor. Although the doctors’ gender did not predict simulated patients’ perceived information exchange and interpersonal relationship building, simulated patients did experience more shared decision making in consultations with female doctors than with male doctors. Therefore, the effect of the doctors’ gender on satisfaction with the consultation is probably because of the difference in perceived shared decision making. This is in line with the meta-analysis of Roter et al [33] that suggests that female doctors engage in more patient-centered communication than male doctors. In addition, because of the focus on gynecological health problems in this study, the female patients in this study may have felt more at ease with a female doctor as they may have a better understanding of their problems and as female gynecologists generally adopt a more patient-centered communication style [57].

Finally, the post hoc analyses showed that the medium of consultation serves as a moderator between communicative behavior and simulated patients’ satisfaction, which implies that the effect of communicative behavior on satisfaction depends on the medium of the consultation while the effects of shared decision making on satisfaction were the same for both types of consultation medium; the effects of perceived information exchange and interpersonal relationship building differed. The effect of perceived information exchange on simulated patients’ satisfaction with the consultation was only significant in the Web-based consultations, whereas the effect of interpersonal relationship building on simulated patients’ satisfaction was only significantly related in the face-to-face consultations. This suggests that simulated patients may expect more information exchange in Web-based consultations and more affective behavior or interpersonal relationship building in face-to-face consultations. The findings of this study suggest that patients especially have lower expectations of affective behavior in Web-based consultations, which is in line with previous studies [45,58]. Patients may see Web-based communication more as a way to share information than to build relationships with their doctor. In addition, the results showed that a less positive attitude toward Web-based communication increased patients’ perceived interpersonal relationship building. There may thus be less actual interpersonal relationship building or affective behavior in screen-to-screen consultations compared with face-to-face consultations, but the lower expectations of Web-based communication can make patients less critical about the shortcomings in Web-based consultations.

### Implications

This study has several implications for both theory and practice. The results of this study provide additional support for the importance of the three main communicative functions of medical interactions for doctors’ and patients’ satisfaction with a consultation. Although several studies have already shown that these behaviors were significantly related to satisfaction in offline consultations [17-19,47,48], this study demonstrates that these three types of communicative behaviors also predict satisfaction with Web-based consultations. Post hoc analyses suggest that information exchange is especially important for satisfaction in Web-based consultations, whereas affective communication is especially important for satisfaction in face-to-face interactions because of the difference in patients’ expectations from face-to-face and Web-based video consultations. In the counseling toward therapy for patients with, for example, pelvic organ prolapse, this is an important outcome, suggesting promising future perspectives for Web-based counseling. It has already been shown that computer-based, Web-based counseling across different clinical settings may improve various health outcomes such as improved glucose control and decreased blood pressure [59], management of urinary incontinence [60], and HIV treatment adherence, and risk reduction for people living with HIV and acquired immune deficiency syndrome [61].

Furthermore, important practical implications can be derived from the findings of this study. As discussed in the introduction, the increase in applications for Web-based doctor-patient interactions elicits worries among doctors and patients as they believe that Web-based consultations can dissocialize and dehumanize the original purpose of doctor-patient interactions, which may, in turn, have a negative impact on the interaction [14,15]. This study shows that these concerns may be unnecessary as there are no differences between screen-to-screen and face-to-face interactions in communicative behavior and satisfaction with the consultation. Other studies have already indicated that Web-based consultations may improve efficiency and reduce the increasing health care costs [62,63]. In addition, previous studies have shown that patients were particularly satisfied with Web-based communication with respect to travel and waiting time, as they could interact with their doctor from their own home at the moment when it is most useful and needed [6,7,16]. Of course, the simulated patients in our study could not profit from those benefits but were still as satisfied with the screen-to-screen as with the face-to-face consultations. These findings underline the potential added value of Web-based video consultations alongside face-to-face consultations as they offer the same interaction quality and satisfaction level as regular face-to-face consultations. Therefore, it is advisable that practitioners and hospitals examine ways to integrate Web-based consultations into their practice. Recent findings showing that physicians with previous experience in using eHealth applications have a more positive attitude toward eHealth implementation and consider that the benefits outweigh its possible difficulties and shortcomings [7,8] are promising in this respect.

### Limitations and Suggestions for Future Research

This study is characterized by a number of strengths and limitations that need to be considered when interpreting the results. First, in this small-scale study, an experimental approach was used in which simulated patients instead of actual patients and interns rather than experienced specialists participated. Although simulated patients ensure high experimental control over the conditions [28,29], they may also be quite similar in characteristics. In this study, all simulated patients were highly educated. Previous research emphasized that less educated patients with low socioeconomic status show low eHealth engagement [64] as they typically have less functional health literacy, which is needed to get information and to understand it, and less critical health literacy, which is needed to critically analyze information and apply it [65]. Therefore, replication with a large and representative sample of nonsimulated patients with different educational levels and different levels of health literacy is needed to confirm our findings.

The participating doctors in this study were young interns who might not yet have a strong preference for a certain type of consultation medium because of their relative lack of experience with medical consultations. Replication with more experienced specialists, who have a long-term experience with regular face-to-face consultations, might yield different results. On the other hand, the absence of a preference for a certain type of consultation medium solely based on experience can also be regarded as a strength of this study as it allows a genuine comparison of consultation medium on the participants’ communicative behavior and satisfaction.

Another limitation is the generalizability of the results. This study focused on gynecological health problems because these types of problems often cause embarrassment. The sensitivity of this topic in particular warrants Web-based communication because of the reduced cues [66]. Furthermore, with gynecology being a leading field in developments of Web-based doctor-patient communication [58,67,68], choosing gynecology was preferable. However, it is necessary to investigate other health care problems as well, as the findings may be different depending on type and phase of the disease. The choice for gynecological health problems also implies that all patients in this study were female. The homogenous group of participants ensured high control over the conditions (and is in that respect a strength of this study); however, it is necessary to investigate whether the same effects count for men as well. To optimally measure shared decision making, this study was set up as a decisional consultation in which the doctor and patient had to make a decision about the treatment. The findings might be different in other types of consultations in which, for example, interpersonal relationship building plays a bigger role. Therefore, replication studies with larger and various patient groups in different phases of their illness will help in determining the generalizability of the results.

Finally, this study focused on doctors’ and patients’ perceived communicative behavior because behavior is most closely related to satisfaction. However, prior research indicates that self-report measures are at risk for social desirability and reporting bias, and therefore, may be inconsistent with actual behavior measures [69]. In addition, the findings of this study suggest that patients may perceive communicative behavior differently in Web-based consultations than in face-to-face consultations, possibly because they may have lower expectations from Web-based communication [45]. Therefore, future research should perform a content analysis to compare doctors’ and patients’ actual communicative behavior during screen-to-screen and face-to-face consultations.

### Conclusions

In conclusion, we found that the quality of doctor-patient communication, as indicated by information exchange, interpersonal relationship building, and shared decision making, did not differ between Web-based and face-to-face consultations. In addition, the results showed that doctors and patients were as satisfied with screen-to-screen consultations as with face-to-face consultations and that there were no differences in the way doctors and patients perceive communicative behavior during these consultations. So far, worries regarding the quality of Web-based medical communication have been a barrier for large-scale implementation of Web-based patient-provider consultations. As this study shows that the interaction quality and satisfaction level are independent of the consultation medium, these results may hopefully offer a step forward in this process. Future studies must be done to demonstrate the efficacy and quality of Web-based medical communication to identify the health outcomes whose benefits appear most promising.

## Appendix

Multimedia Appendix 1 Flowchart of experimental consultation sessions.

Multimedia Appendix 2 Extra explanation of the operationalization of the mediating and dependent variables.

## Abbreviations

CMC: computer-mediated communication
eHealth: electronic health
LEAPS: Learn, Educate, Assess, Partner and Support
PPS: Patient Participation Scale
PSQ: Patient Satisfaction Questionnaire
SIP: Social Information Processing

## References

[1] Kreps GL, Neuhauser L (2010). New directions in eHealth communication: opportunities and challenges. Patient Educ Couns.

[2] Buntin MB, Burke MF, Hoaglin MC, Blumenthal D (2011). The benefits of health information technology: a review of the recent literature shows predominantly positive results. Health Aff (Millwood).

[3] Knight P, Bonney A, Teuss G, Guppy M, Lafferre D, Mullan J, Barnett S (2016). Positive clinical outcomes are synergistic with positive educational outcomes when using telehealth consulting in general practice: a mixed-methods study. J Med Internet Res.

[4] Elbert NJ, van Os-Medendorp H, van Renselaar W, Ekeland AG, Hakkart-van Rooijen L, Raat H, Nijsten TE, Pasmans SG (2014). Effectiveness and cost-effectiveness of ehealth interventions in somatic diseases: a systematic review of systematic reviews and meta-analyses. J Med Internet Res.

[5] Black AD, Car J, Pagliari C, Anandan C, Cresswell K, Bokun T, McKinstry B, Procter R, Majeed A, Sheikh A (2011). The impact of eHealth on the quality and safety of health care: a systematic overview. PLoS Med.

[6] Peeters JM, Krijgsman JW, Brabers AE, Jong JD, Friele RD (2016). Use and uptake of eHealth in general practice: a cross-sectional survey and focus group study among health care users and general practitioners. JMIR Med Inform.

[7] Ariens LF, Schussler-Raymakers FM, Frima C, Flinterman A, Hamminga E, Arents BW, Bruijnzeel-Koomen CA, de Bruin-Weller MS, van Os-Medendorp H (2017). Barriers and facilitators to eHealth use in daily practice: perspectives of patients and professionals in dermatology. J Med Internet Res.

[8] Ruiz Morilla MD, Sans M, Casasa A, Giménez N (2017). Implementing technology in healthcare: insights from physicians. BMC Med Inform Decis Mak.

[9] Hersh WR, Helfand M, Wallace J, Kraemer D, Patterson P, Shapiro S, Greenlick M (2001). Clinical outcomes resulting from telemedicine interventions: a systematic review. BMC Med Inform Decis Mak.

[10] Weiner JP, Yeh S, Blumenthal D (2013). The impact of health information technology and e-health on the future demand for physician services. Health Aff (Millwood).

[11] Haluza D, Jungwirth D (2015). ICT and the future of health care: aspects of health promotion. Int J Med Inform.

[12] Lupiáñez-Villanueva F, Hardey M, Torrent J, Ficapal P (2010). The integration of Information and Communication Technology into medical practice. Int J Med Inform.

[13] Illiger K, Hupka M, von Jan U, Wichelhaus D, Albrecht U (2014). Mobile technologies: expectancy, usage, and acceptance of clinical staff and patients at a university medical center. JMIR Mhealth Uhealth.

[14] Turner JW, Thomas RJ, Reinsch NL (2004). Willingness to try a new communication technology: perpetual factors and task situations in a health care context. Int J Bus Commun.

[15] Matusitz J, Breen G (2007). Tandfonline.

[16] Mair F, Whitten P (2000). Systematic review of studies of patient satisfaction with telemedicine. Br Med J.

[17] Ong LM, De Haes JC, Hoos AM, Lammes FB (1995). Doctor-patient communication: a review of the literature. Soc Sci Med.

[18] Bensing J, van Dulmen S, Tates K (2003). Communication in context: new directions in communication research. Patient Educ Couns.

[19] de Haes H, Bensing J (2009). Endpoints in medical communication research, proposing a framework of functions and outcomes. Patient Educ Couns.

[20] Antheunis ML, Valkenburg PM, Peter J (2007). Computer-mediated communication and interpersonal attraction: an experimental test of two explanatory hypotheses. Cyberpsychol Behav.

[21] Antheunis ML, Schouten AP, Valkenburg PM, Peter J (2011). Interactive uncertainty reduction strategies and verbal affection in computer-mediated communication. Communic Res.

[22] Liu X, Sawada Y, Takizawa T, Sato H, Sato M, Sakamoto H, Utsugi T, Sato K, Sumino H, Okamura S, Sakamaki T (2007). Doctor-patient communication: a comparison between telemedicine consultation and face-to-face consultation. Intern Med.

[23] Elwyn G, Frosch D, Thomson R, Joseph-Williams N, Lloyd A, Kinnersley P, Cording E, Tomson D, Dodd C, Rollnick S, Edwards A, Barry M (2012). Shared decision making: a model for clinical practice. J Gen Intern Med.

[24] Ilioudi S, Lazakidou A, Tsironi M (2010). Information and communication technologies for better patient self-management and self-efficacy. Int J Electron Healthc.

[25] Barry MJ, Edgman-Levitan S (2012). Shared decision making--pinnacle of patient-centered care. N Engl J Med.

[26] Wu JM, Matthews CA, Conover MM, Pate V, Jonsson Funk M (2014). Lifetime risk of stress urinary incontinence or pelvic organ prolapse surgery. Obstet Gynecol.

[27] Lane C, Rollnick S (2007). The use of simulated patients and role-play in communication skills training: a review of the literature to August 2005. Patient Educ Couns.

[28] Glassman PA, Luck J, O'Gara EM, Peabody JW (2000). Using standardized patients to measure quality: evidence from the literature and a prospective study. Jt Comm J Qual Patient Saf.

[29] Patel B, Johnston M, Cookson N, King D, Arora S, Darzi A (2016). Interprofessional communication of clinicians using a mobile phone app: a randomized crossover trial using simulated patients. J Med Internet Res.

[30] Nederlandse Zorgautoriteit (Dutch Healthcare Authority) (2013) NZA.

[31] Abrams P, Andersson KE, Birder L, Brubaker L, Cardozo L, Chapple C, Cottenden A, Davila W, de Ridder D, Dmochowski R, Drake M, Dubeau C, Fry C, Hanno P, Smith JH, Herschorn S, Hosker G, Kelleher C, Koelbl H, Khoury S, Madoff R, Milsom I, Moore K, Newman D, Nitti V, Norton C, Nygaard I, Payne C, Smith A, Staskin D, Tekgul S, Thuroff J, Tubaro A, Vodusek D, Wein A, Wyndaele JJ, Members OC, Fourth International Consultation on Incontinence (2010). Fourth international consultation on incontinence recommendations of the International Scientific Committee: evaluation and treatment of urinary incontinence, pelvic organ prolapse, and fecal incontinence. Neurourol Urodyn.

[32] Hirsch O, Keller H, Albohn-Kühne C, Krones T, Donner-Banzhoff N (2011). Pitfalls in the statistical examination and interpretation of the correspondence between physician and patient satisfaction ratings and their relevance for shared decision making research. BMC Med Res Methodol.

[33] Roter DL, Wexler R, Naragon P, Forrest B, Dees J, Almodovar A, Wood J (2012). The impact of patient and physician computer mediated communication skill training on reported communication and patient satisfaction. Patient Educ Couns.

[34] Ommen O, Thuem S, Pfaff H, Janssen C (2011). The relationship between social support, shared decision-making and patient's trust in doctors: a cross-sectional survey of 2,197 inpatients using the Cologne Patient Questionnaire. Int J Public Health.

[35] Pfaff H, Freise D, Mager G, Schrappe M (2003). Der Kölner Patientenfragebogen (KPF): Entwicklung und Validierung eines Fragebogens zur Erfassung der Einbindung des Patienten als Kotherapeuten.

[36] Blanchard CG, Ruckdeschel JC, Fletcher BA, Blanchard EB (1986). The impact of oncologists' behaviors on patient satisfaction with morning rounds. Cancer.

[37] Ong LM, Visser MR, Lammes FB, De Haes JC (2000). Doctor-patient communication and cancer patients' quality of life and satisfaction. Patient Educ Couns.

[38] Zandbelt L, Smets E, Oort F, Godfried M, De Haes H (2004). Satisfaction with the outpatient encounter. J Gen Intern Med.

[39] Yen C, Tu C (2008). Online social presence: a study of score validity of the computer-mediated communication questionnaire. QRDE.

[40] Preacher KJ, Hayes AF (2008). Asymptotic and resampling strategies for assessing and comparing indirect effects in multiple mediator models. Behav Res Methods.

[41] Baron RM, Kenny DA (1986). The moderator–mediator variable distinction in social psychological research: conceptual, strategic, and statistical considerations. J Pers Soc Psychol.

[42] Walther JB (1992). Interpersonal effects in computer-mediated interaction: a relational perspective. Communic Res.

[43] Walther JB (2005). Let me count the ways: the interchange of verbal and nonverbal cues in computer-mediated and face-to-face affinity. J Lang Soc Psychol.

[44] Roter DL, Frankel RM, Hall JA, Sluyter D (2006). The expression of emotion through nonverbal behavior in medical visits. Mechanisms and outcomes. J Gen Intern Med.

[45] Agha Z, Roter DL, Schapira RM (2009). An evaluation of patient-physician communication style during telemedicine consultations. J Med Internet Res.

[46] Han WT, Collie K, Koopman C, Azarow J, Classen C, Morrow GR, Michel B, Brennan-O'Neill E, Spiegel D (2005). Breast cancer and problems with medical interactions: relationships with traumatic stress, emotional self-efficacy, and social support. Psychooncology.

[47] Arora NK (2003). Interacting with cancer patients: the significance of physicians' communication behavior. Soc Sci Med.

[48] Lee SJ, Back AL, Block SD, Stewart SK (2002). Enhancing physician-patient communication. Hematology Am Soc Hematol Educ Program.

[49] Engel GL (1992). How much longer must medicine's science be bound by a seventeenth century world view?. Psychother Psychosom.

[50] Kiesler DJ, Auerbach SM (2006). Optimal matches of patient preferences for information, decision-making and interpersonal behavior: evidence, models and interventions. Patient Educ Couns.

[51] O'Connor AM, Drake ER, Wells GA, Tugwell P, Laupacis A, Elmslie T (2003). A survey of the decision-making needs of Canadians faced with complex health decisions. Health Expect.

[52] Faller H (2003). [Shared decision making: an approach to strengthening patient participation in rehabilitation]. Rehabilitation (Stuttg).

[53] Farin E (2010). [Patient-provider communication in chronic illness: current state of research in selected areas]. Rehabilitation (Stuttg).

[54] Quaschning K, Körner M, Wirtz M (2013). Analyzing the effects of shared decision-making, empathy and team interaction on patient satisfaction and treatment acceptance in medical rehabilitation using a structural equation modeling approach. Patient Educ Couns.

[55] Charles CA, Whelan T, Gafni A, Willan A, Farrell S (2003). Shared treatment decision making: what does it mean to physicians?. J Clin Oncol.

[56] Hamann J, Mendel R, Bühner M, Kissling W, Cohen R, Knipfer E, Eckstein H (2012). How should patients behave to facilitate shared decision making--the doctors' view. Health Expect.

[57] Janssen SM, Lagro-Janssen AL (2012). Physician's gender, communication style, patient preferences and patient satisfaction in gynecology and obstetrics: a systematic review. Patient Educ Couns.

[58] Aarts JWM, van Oers AM, Faber MJ, Cohlen BJ, Nelen WL, Kremer JA, van Dulmen AM (2015). Communication at an online infertility expert forum: provider responses to patients' emotional and informational cues. J Psychosom Obstet Gynaecol.

[59] Flodgren G, Rachas A, Farmer AJ, Inzitari M, Shepperd S (2015). Interactive telemedicine: effects on professional practice and health care outcomes. Cochrane Database Syst Rev.

[60] Hui E, Lee PS, Woo J (2006). Management of urinary incontinence in older women using videoconferencing versus conventional management: a randomized controlled trial. J Telemed Telecare.

[61] Kurth AE, Chhun N, Cleland CM, Crespo-Fierro M, Parés-Avila JA, Lizcano JA, Norman RG, Shedlin MG, Johnston BE, Sharp VL (2016). Linguistic and cultural adaptation of a computer-based counseling program (CARE+ Spanish) to support HIV treatment adherence and risk reduction for people living with HIV/AIDS: a randomized controlled trial. J Med Internet Res.

[62] Ahern DK, Kreslake JM, Phalen JM (2006). What is eHealth (6): perspectives on the evolution of eHealth research. J Med Internet Res.

[63] Harrison JP, Lee A (2006). The role of e-Health in the changng health care environment. Nurs Econ.

[64] Kontos E, Blake KD, Chou WS, Prestin A (2014). Predictors of eHealth usage: insights on the digital divide from the Health Information National Trends Survey 2012. J Med Internet Res.

[65] Nutbeam D (2008). The evolving concept of health literacy. Soc Sci Med.

[66] Walther JB (2016). Computer-Mediated Communication. Communic Res.

[67] Haggerty AF, Huepenbecker S, Sarwer DB, Spitzer J, Raggio G, Chu CS, Ko E, Allison KC (2016). The use of novel technology-based weight loss interventions for obese women with endometrial hyperplasia and cancer. Gynecol Oncol.

[68] Bientzle M, Griewatz J, Kimmerle J, Küppers J, Cress U, Lammerding-Koeppel M (2015). Impact of scientific versus emotional wording of patient questions on doctor-patient communication in an internet forum: a randomized controlled experiment with medical students. J Med Internet Res.

[69] Tachakra S, Rajani R (2002). Social presence in telemedicine. J Telemed Telecare.

