# Supplementary material for: The Effect of Screen-to-Screen Versus Face-to-Face Consultation on Doctor-Patient Communication: An Experimental Study with Simulated Patients
Source: J Med Internet Res. 2017 Dec 20;19(12):e421. doi: 10.2196/jmir.8033 (PMC5752968; doi:10.2196/jmir.8033)
Supplement: Multimedia Appendix 1 [file jmir_v19i12e421_app1.pdf]

Appendix 1: Flowchart experimental consultation sessions

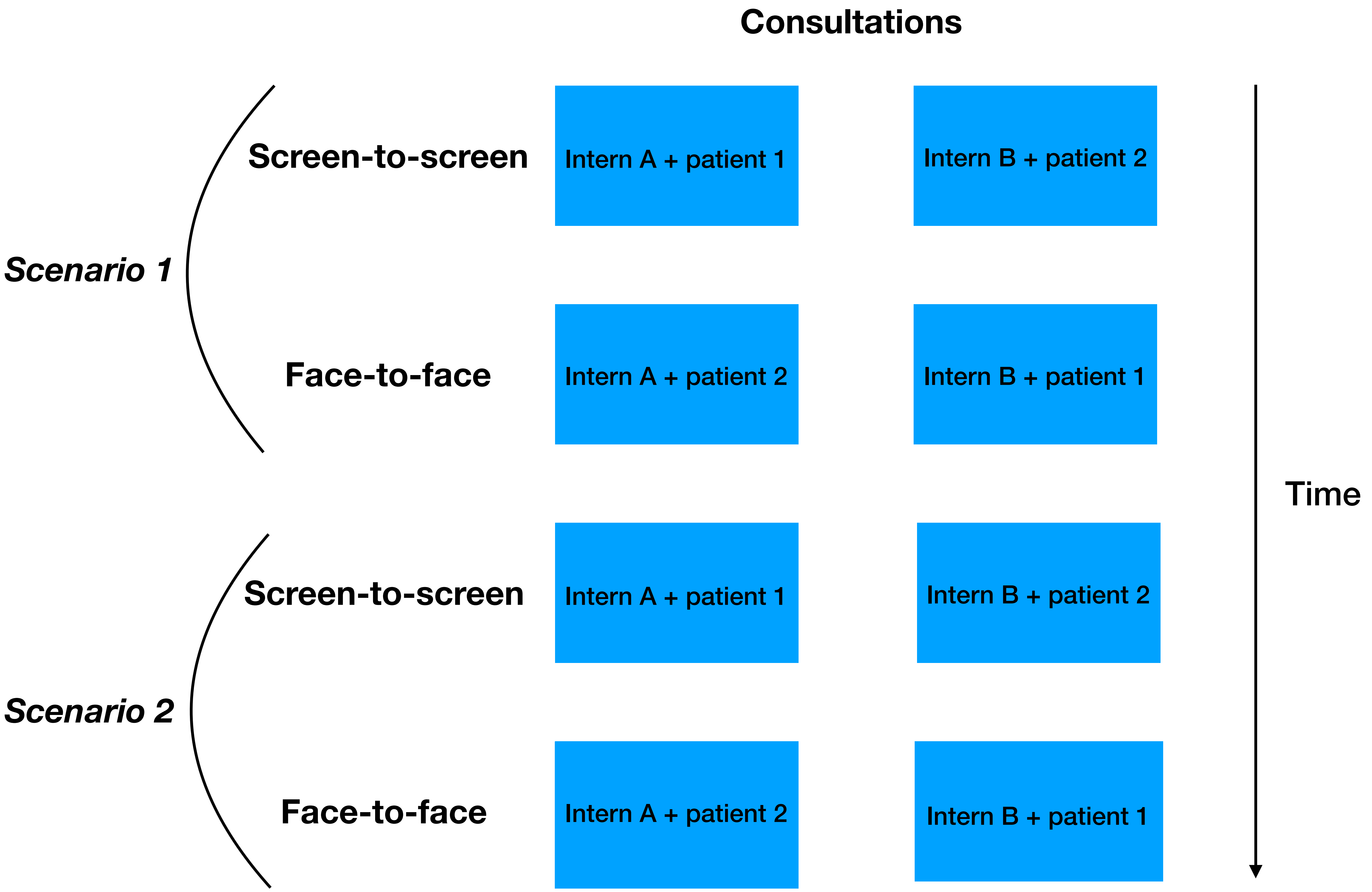

This is an example of a session, there were a total of six sessions. In each session participated 2 interns and 2 simulated patients.
