# Supplementary material for: The Effect of Screen-to-Screen Versus Face-to-Face Consultation on Doctor-Patient Communication: An Experimental Study with Simulated Patients
Source: J Med Internet Res. 2017 Dec 20;19(12):e421. doi: 10.2196/jmir.8033 (PMC5752968; doi:10.2196/jmir.8033)
Supplement: Multimedia Appendix 2 [file jmir_v19i12e421_app2.pdf]

Extra explanation of the operationalization of the mediating and dependent variables

Perceived Information Exchange

Five items were used to measure patients' perceived information exchange. The first 3 items belong to the *Patient Participation Scale (PPS) [32]*: "My doctor helped me to understand all the information", "My doctor understood what is important for me", and "My doctor answered all of my questions". Since the *PPS* only takes doctors' behavior into account, 2 items of the *LEAPS Framework (Learn, Educate, Assess, Partner and Support)* [33], from the subparts *information exchange* and *identification of problems and concerns,* were added in order to measure patients' behavior. The items were: "I asked for an explanation of medical terms I didn't know", and "I checked that the doctor clearly understood the information that I gave him/her". The response categories for all items ranged from 1 (*completely disagree*) to 5 (*completely agree*). All items formed a one-dimensional scale (explained variance 50%, alpha=.73, mean 3.49 [SD 0.58]).

Doctors' perceived information exchange was measured by the same 3 items from the *PPS* [32] as in the patients' questionnaire, adapted to fit the doctors' perspective. In addition, 3 items from the subparts *information exchange* and *identification of problems and concerns* of the *LEAPS Framework* [33] were added: "The patient had difficulty remembering instructions", "The patient did not understand my explanations of the medical problem and treatment", and "I could not understand all the patient wanted to tell me". The response categories were the same as used in the patient version. The 6 items formed a one-dimensional scale (explained variance 45%, alpha=.73, mean 4.13 [SD 0.41]).

Perceived Interpersonal Relationship Building

To measure patients' perceived interpersonal relationship building, 5 items were used. The first 2 items measured doctors' affective behavior and were derived from the subpart *patients' evaluation of emotional support of the physician* from the *Cologne Patient Questionnaire* [34]. The items were: "It was possible to talk with the doctor about personal matters", and "The doctor carried out the conversation with me in a very empathetic manner". The other 3 items were related to patients' self-disclosure and were derived from the subpart *interpersonal rapport* of the *LEAPS Framework* [33]: "I let the doctor know when I felt worried about my condition or treatment", "I told the doctor about my health worries and concerns", and "The doctor did not do a good job addressing my fears and concerns". For all 5 items, the response

categories ranged from 1 (*completely disagree*) to 5 (*completely agree*). The items formed a one-dimensional scale (explained variance 50%, alpha=.73, mean 3.80 [SD 0.49]).

To measure doctors' perceived interpersonal relationship building, the 2 items of the *Cologne Patient Questionnaire* [34,35] which were used to measure patients' perceived interpersonal relationship formation were adapted to the doctors' perspective; "I made it possible for the patient to talk about personal matters", and "I carried out the conversation with the patient in a very empathic manner". In addition, 3 items of the subpart *interpersonal rapport* of the *LEAPS Framework* [33] were used; "I have trouble connecting emotionally with this patient", "I communicated empathy to the patient", and "I legitimated worries and concerns". For all 5 items, the response categories ranged from 1 (*completely disagree*) to 5 (*completely agree*). The items formed a one-dimensional scale (explained variance 47%, alpha=.67, mean 3.84 [SD 0.39]).

Perceived Shared Decision Making

Patients' shared decision making was measured using 7 items: 3 items of the *PPS* [32], and 4 items of the *Cologne Patient Questionnaire* [34,35], from the subpart *patients' evaluation of shared decision making behavior of the physician.* Example items are: "I was sufficiently involved in decisions about my treatment", and "The doctor wanted me to be actively involved in the treatment process". The response categories ranged from 1 (completely disagree) to 5 (completely agree). The 7 items formed a one-dimensional scale (explained variance 64%, alpha=.89, mean 4.02 [SD 0.63]).

The 7 items which measured patients' shared decision-making were adapted to measure doctors' shared decision-making, by turning the patient's perspective to the doctor's perspective. "I was sufficiently involved in decisions about the treatment" was for example changed in "I sufficiently involved the patient in decisions about the treatment". The 7 items formed a one-dimensional scale (explained variance 54%, alpha=.83, mean 4.08 [SD 0.49]).

Satisfaction With the Consultation

Patients' satisfaction with the consultation was measured using the *Patient Satisfaction Questionnaire (PSQ) [36,37]*, which consists of 5 items measuring patients' satisfaction with the way their needs were addressed, their active involvement in the interaction, information received, emotional support received, and the interaction in general. Two items of the *PSQ* were adapted so they would measure satisfaction with certain behavior instead of behavior itself. For example "How well did the doctor address your needs" was changed into "How satisfied are you with the way the doctor addressed your needs". In addition, one additional item was added to the scale which measured satisfaction regarding the treatment decision, as making a decision

regarding the treatment was the purpose of the consultations in this experiment. All items were answered on a scale from 1 (*not at all satisfied*) to 5 *(extremely satisfied)*. The 6 items formed a one-dimensional scale (explained variance 53%, alpha=.85, mean 4.33 [SD 0.42]).

To measure doctors' satisfaction with the consultation, the items of the *PSQ* and the item measuring satisfaction with the treatment decision were adapted to the doctors' situation as suggested by Zandbelt and colleagues [38]. For example, the item "How well did the doctor address your needs" was modified to "How well did you address the needs of this patient". The response categories were the same as in the patient version. The 6 items formed a one-dimensional scale (explained variance 48%, alpha=.76, mean 4.29 [SD 0.39]).
